# Dual-Band Circularly Polarized Dielectric Resonator Antenna for WLAN and WiMAX Applications

**DOI:** 10.3390/s20041137

**Published:** 2020-02-19

**Authors:** Amir Altaf, Munkyo Seo

**Affiliations:** Department of Electrical and Computer Engineering, Sungkyunkwan University, Suwon 440-746, Korea; amiraltaf@skku.edu

**Keywords:** dielectric resonator antenna, dual-band circular polarization, dual-sense circular polarization, single-point feeding

## Abstract

In this paper, a new dual-band circularly polarized (CP) dielectric resonator antenna for WLAN and WiMAX applications is proposed. The dielectric resonator has an asymmetric Y-shaped geometry. By properly selecting the length of the arms, the pairs of fundamental (TE${}_{111}$) and second-order (TE${}_{211}$) are excited separately at the design frequencies to radiate the CP wave. The measurement shows that the fabricated antenna exhibits a wide impedance bandwidth for |S${}_{11}|<$−10 dB of 62.07% (2.2–4.18 GHz). The far-field measurements in the broadside direction demonstrated a dual-band CP response with 3 dB ARBWs of 4.92%(2.38–2.5 GHz) at the lower and 12.64% (3.26–3.70 GHz) at the upper band. The measured CP bands cover the entire frequency range of WLAN (2.401–2.495 GHz) and WiMAX (3.4–3.69 GHz) at the lower and upper bands, respectively.

## 1. Introduction

Dielectric resonator antennas (DRAs) are the ceramic antennas that become efficient radiators upon proper excitation. They are preferred over metallic antennas due to their high radiation efficiency (generally ≥ 90%), as no metallic losses are present. Based on the radiations from the DRA, the circularly polarized (CP) radiators are given preference over the linearly polarized (LP) radiators as they are resistant to multi-path interference and fadings, and insensitive to the orientation of the transmitter and receiver antennas.

In literature, the CP radiation from the DRA arises either by selecting an optimum feeding method or by modifying the dielectric resonator (DR) geometry to ensure the simultaneous excitation of two degenerate modes that are of the same magnitude and 90° apart in phase. To reduce the design complexity and ensure compactness, the single-point feeding is favored over the dual-point feeding. Recently, there is an interest in developing single-point fed dual-band CP DRAs to manage the demands of modern communication systems. Several DRAs have been demonstrated that excite the orthogonal pairs of fundamental and higher-order modes to produce dual-band circular polarization [1,2,3,4,5,6,7,8,9]. For instance, a grooved DR with corner truncation is designed that has a dual-band CP response [1]. In one study, a fan-blade shaped DR radiate dual-band CP wave [2]. In another study, a triangular aperture coupled DRA with a dual-band circular polarization is presented [3]. Similarly, an inverted-sigmoid-shaped DR is designed by Varshney at el. that provides circular polarization in two different frequency range [4]. The other dual-band CP DRAs are the examples of hybrid antennas that employ orthogonal modes of the DR and slot/patch to yield dual-band CP radiation [10,11,12]. From these designs [10,12] utilized the slot modes and [11] utilized the patch mode to produce circular polarization at the lower band, while the upper CP bands in all designs are produced by the DR.

In literature, both directional [13,14] and omni-directional [15,16] antennas have been developed for Wireless Local Area Network (WLAN) and Wireless Interoperability for Microwave Access (WiMAX) applications. The selection of the appropriate antenna depends on the intended application. For instance, in case of point-to-point or nearly line-of-sight applications, directional antennas are preferred whereas in case of access point/routers omni-directional antennas are used. In this paper, a novel directional dual-band CP DRA is designed for WLAN and WiMAX applications. The CP radiation at the desired frequency bands is achieved owing to the geometry of the DR, which produces the degenerate modes of quadrature phase-difference and equal magnitudes. The lower CP band arises from the excitation of the fundamental mode, while the second-order mode is dominant at the upper CP band. Three DR geometries that are named as DR-1, DR-2, and proposed DR are utilized as radiators over the same ground plane. The axial ratio (AR) comparison concludes that only the proposed DR radiates dual-band CP waves. The antenna design, parametric study, and comparison of the measurements with the simulation are described below in separate sections.

## 2. Antenna Configuration

**Figure 1:**
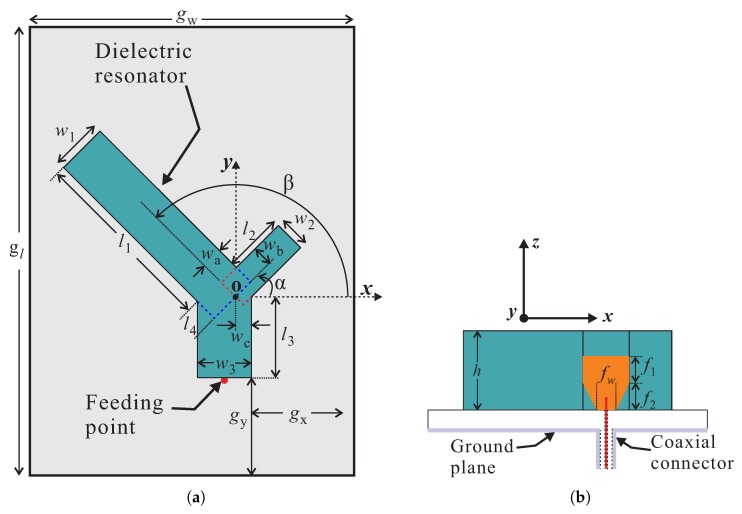
Geometry of the proposed antenna: (**a**) Top view; (**b**) Side view.

Figure 1 represents the geometry of the proposed dual-band CP DRA. An asymmetric Y-Shaped DR ($\varepsilon_{dr}$ = 10) is placed on top of a 1.52-mm thick Taconic RF-35 substrate ($\varepsilon_{r}$ = 3.5). The lower side of the substrate is fully covered with a ground plane of dimensions $g_{w}\times g_{l}$. The Y-shaped DR is created by combining three rectangular arms—long, medium, and short—with each of a height *h*. The long arm has a length and width of $l_{1}+l_{4}$ and $w_{1}$, respectively. The medium and short arms are of lengths $l_{2}+w_{a}$ and $l_{3}$ and widths $w_{2}$ and $w_{3}$, respectively. Considering the origin at “o”, the medium and long arms are rotated by α and β, respectively. The DR is placed at a distance of $g_{x}$ and $g_{y}$ from the lower right corner of the substrate. A single-point feeding mechanism that employs a vertical-strip is attached to the short arm of the DR for excitation. The vertical-strip has a length of $f_{1}+f_{2}$ where the lower end is tapered by dimensions of $\left(w_{3}-f_{w}\right)\times f_{2}$. The optimized geometric parameters are mentioned in Table 1.

**Figure 2:**
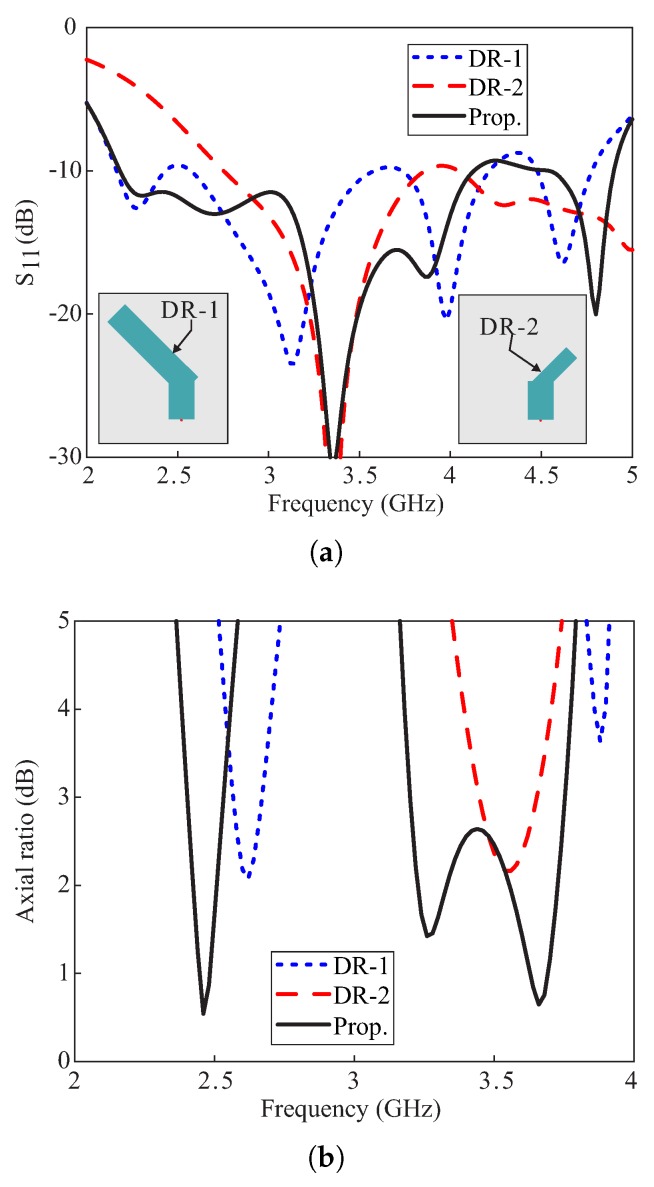
Comparison of the simulated results of the antenna designs with DR-1, DR-2, and proposed DRs as radiators: (**a**) Reflection coefficients; (**b**) Axial ratios.

Figure 2 compares the simulated reflection coefficients and ARs of three antennas that utilize the geometries of DR-1, DR-2, and proposed DR as radiators. The performance of these antennas is evaluated by considering −10 dB IBW and 3 dB ARBW criteria. The geometries of DR-1 and DR-2 are depicted in Figure 2a and are designed by removing the medium and long arms from the proposed DR, respectively. Also, the lengths of $l_{4}$ and $w_{a}$ are extended to exclude the medium discontinuity in the design of DR-1 and DR-2, respectively. From Figure 2a, DR-1 based antenna design yields a multi-band response. Since the size of DR-2 is smaller than DR-1, the DR-2 based design resonates near 3.4 GHz and an overall dual-band response is obtained. The second and third harmonics in Figure 2a for the DR-1 based design are due to the separate excitation of TE${}_{131}$ and quasi-TE${}_{141}$ modes, respectively. Similarly, the upper band for the DR-2 based antenna shows a TE${}_{212}$-like mode at the center frequency of 4.62 GHz. The proposed DR based antenna has a dual-band response, where the lower band is wider as compared to the other two designs. To achieve circular polarization at a certain frequency with AR ≤3 dB, the $E_{x}$ and $E_{y}$ components of the E-field should have an amplitude ratio of ≤$\frac{1}{\sqrt{2}}$ and a phase deviation of ≤35.26 around ±90° [17]. Figure 2b compares the ARs of the three designs. It can be observed that DR-1 based antenna radiates a CP wave around 2.62 GHz and an elliptically polarized wave around 3.88 GHz. Moreover, the CP wave at 2.62 GHz is right-handed circularly polarized (RHCP), while the elliptically polarized band has a left-handed sense of rotation. For DR-2 based design, a narrow CP band is obtained around 3.56 GHz that is left-handed circularly polarized (LHCP). The sense of CP wave radiated from DR-1 and DR-2 based designs can be easily determined by plotting the E-field distributions and are not shown here for brevity. Unlike DR-1 and DR-2 based antennas, the proposed Y-shaped DR based design possesses dual-band circular polarization. Compared to DR-1 based design, the CP band is not only shifted downwards in frequency but the quality of the CP wave has also improved. The upper CP band of the proposed antenna is a combination of a CP band of DR-2 and an elliptically polarized band of DR-1 based designs that are merged to produce a wide 3 dB ARBW. Additionally, the lower −10 dB band of the proposed DR covers both of the CP bands, therefore, only this band will be concentrated throughout the remaining manuscript.

**Figure 3:**
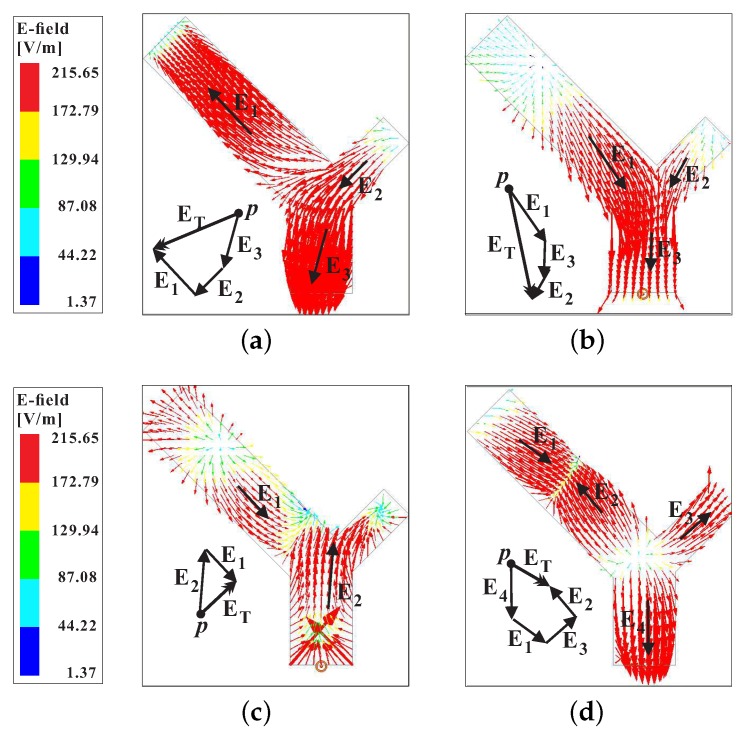
Simulated E-field on the top surface of the proposed Y-shaped DR within the CP bands: (**a**) 46 GHz at *t*= 0; (**b**) 2.46 GHz at *t* = *T*/4; (**c**) 3.5 GHz at *t* = 0; (**d**) 3.5 GHz at *t* = *T*/4.

**Figure 4:**
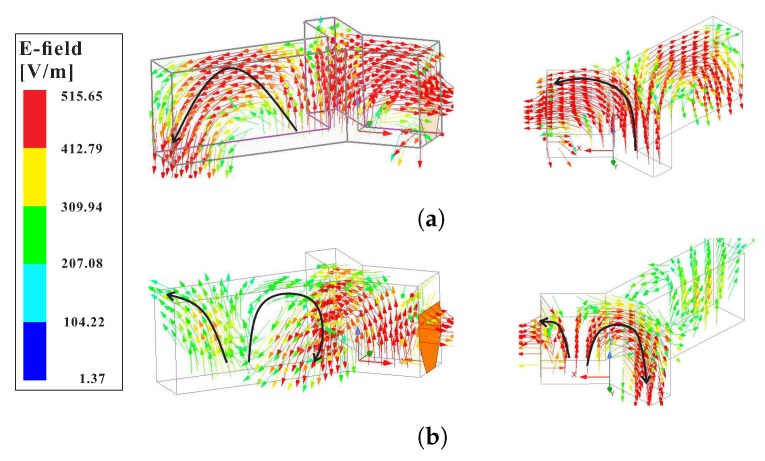
Mode distributions inside the proposed Y-shaped DR at the CP bands: (**a**) Quasi-TE${}_{111}$ at 2.46 GHz; (**b**) Quasi-TE${}_{211}$ at 3.5 GHz.

The sense of circular polarization at both CP bands can be determined by observing the rotation of a resultant E-field vector $\vec{E_{T}}$ with a change in time ‘t’ from *t* = 0 to *t* = *T*/4. Figure 3 plots the E-field vectors $\vec{E_{i}}$, where *i* = 1, …, 4, at the top surface of the proposed Y-shaped DR. Considering the origin at point *p*, the vector sum $\vec{E_{T}}$ in Figure 3a,b, lies in the third and fourth quadrants, respectively. These vector sums are orthogonal to each other and rotate in an anti-clockwise direction as *t* goes from *t* = 0 to *t* = *T*/4, thereby producing a RHCP. For the upper CP band, the E-field is plotted at 3.5 GHz for *t* = 0 and *t* = *T*/4 as shown in Figure 3c,d, respectively. It can be seen that for a change in time Δt= *T*/4, the vector sum $\vec{E_{T}}$ rotates in a clockwise direction from first to fourth quadrant and radiate a wave with a LHCP sense of rotation. Furthermore, the sense of CP at both bands can be simultaneously changed by mirroring the whole antenna structure along the −y-axis. However, the sense of CP at an individual band cannot be altered separately. As discussed earlier, the orthogonal modes of the same magnitude and 90° out of phase contribute to the excitation of circular polarization in the antenna. Figure 4 depicts the E-field distribution of the modes at 2.46 GHz and 3.5 GHz, respectively. It is obvious from Figure 4a, that the long arm of the Y-shaped DR operates in the fundamental modes. Also, the combination of medium and short arms produces a fundamental mode distribution. At 3.5 GHz, the long arm alone and a combination of medium and short arms have field distributions that are similar to the quasi-TE${}_{211}$ mode.

## 3. Parametric Analysis

The simulations for the parametric study were carried out in HFSS software. In these simulations, the effect of variations in $l_{1}$, $l_{2}$, and β on the reflection coefficients and ARs are analyzed. Also, only the parameter of interest was varied during the simulation while all others were fixed.

**Figure 5:**
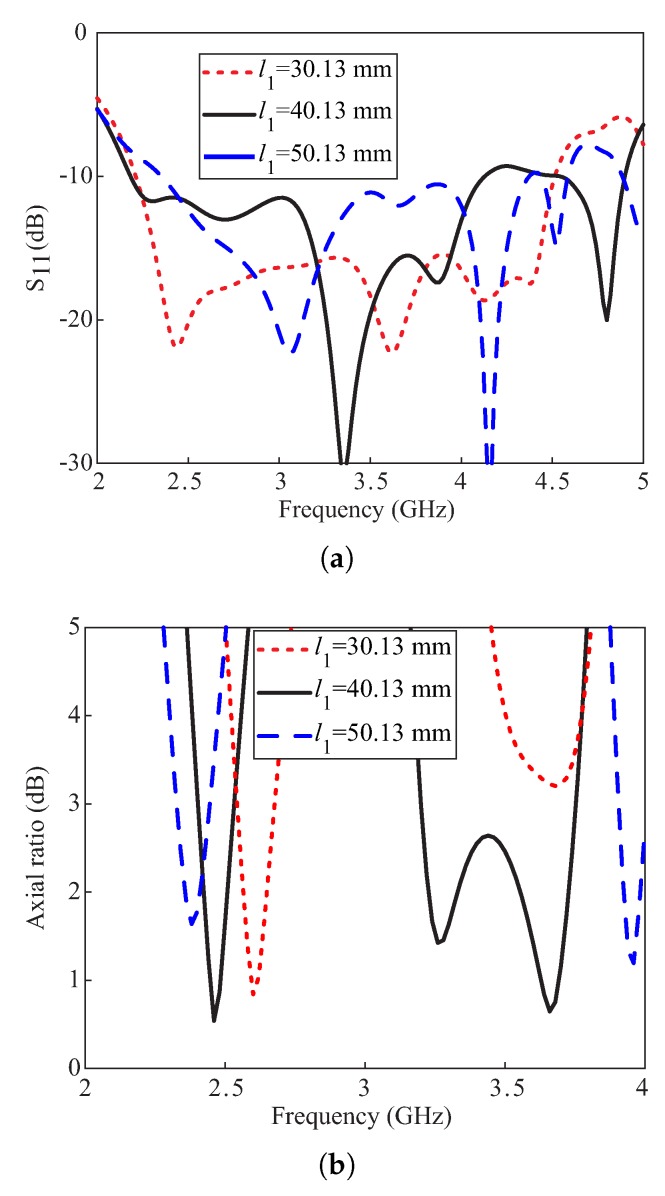
Simulated effects of varying the length $l_{1}$ of the DR: (**a**) Reflection coefficients; (**b**) Axial ratios.

The simulated effects of varying the length $l_{1}$ of the DR on the reflection coefficients and ARs are plotted in Figure 5. From the results shown in Figure 5a, it is observed that the variations in $l_{1}$ affect the reflection coefficients at each frequency point, and a widest −10 dB IBW is obtained for $l_{1}$ = 30.13 mm. The AR performance of the proposed antenna varies with variations in length $l_{1}$ as depicted in Figure 5b. The lower CP band shows a trend of shifting towards lower frequencies with an increase in values of $l_{1}$. The upper CP band is highly sensitive to this variation and ARs ≤ 3 dB are obtained only for $l_{1}$ = 40.13 mm and $l_{1}$ = 50.13 mm. The chosen value of $l_{1}$ = 40.13 mm based on the wide 3 dB ARBW performance.

**Figure 6:**
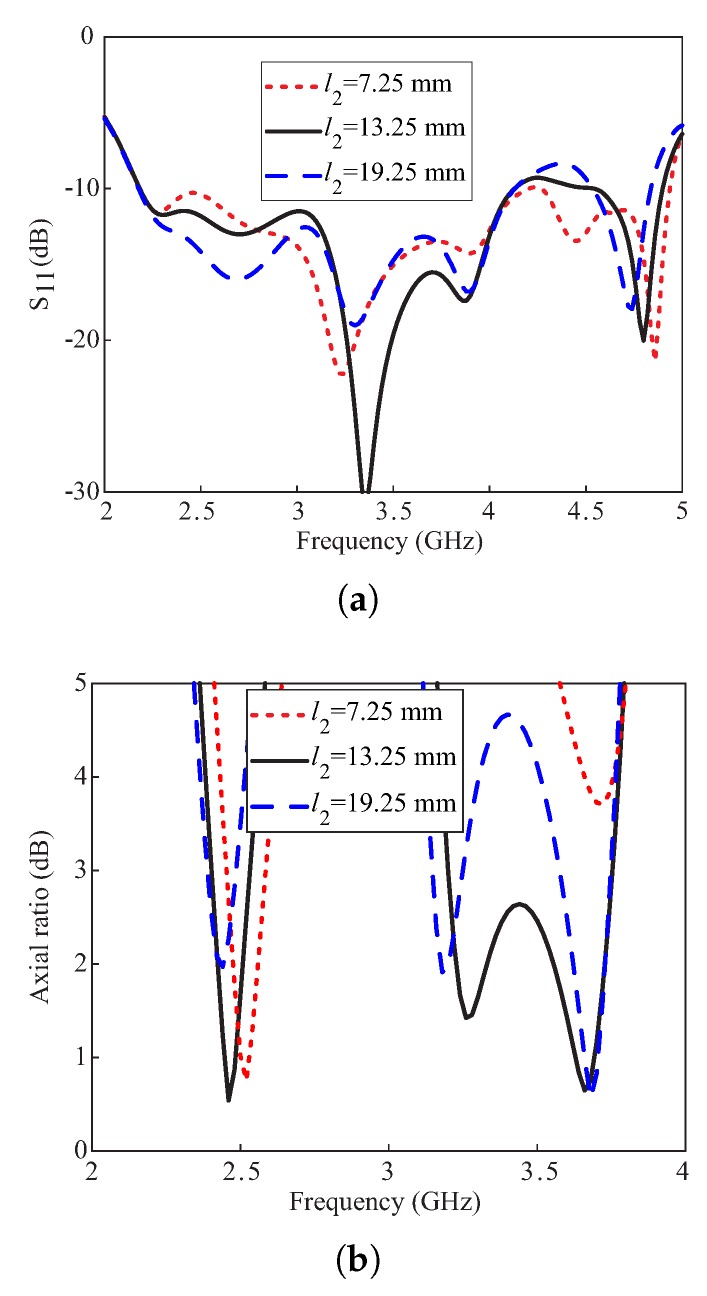
Simulated effects of varying the length $l_{2}$ of the DR: (**a**) Reflection coefficients; (**b**) Axial ratios.

Figure 6 represents the simulated effects on the reflection coefficients and ARs with a change in length $l_{2}$ of the DR. From Figure 6a, the −10 dB IBW remains insensitive to the variations in $l_{2}$. From Figure 6b, a narrow 3 dB ARBW is obtained at the lower band that is shifting towards lower frequencies as $l_{2}$ is increased. For the upper band, a wide 3 dB ARBW is produced only for $l_{2}$ = 13.25 mm and two narrow CP bands for $l_{2}$ =19.25 mm. It is also noticed that the AR minimum around 3.2 GHz at the upper CP band is shifted downwards in frequency with an increase in the value of $l_{2}$, while the upper minimum remains almost constant. This concludes that the $l_{2}$ contributes to the generation of CP around 3.2 GHz.

**Figure 7:**
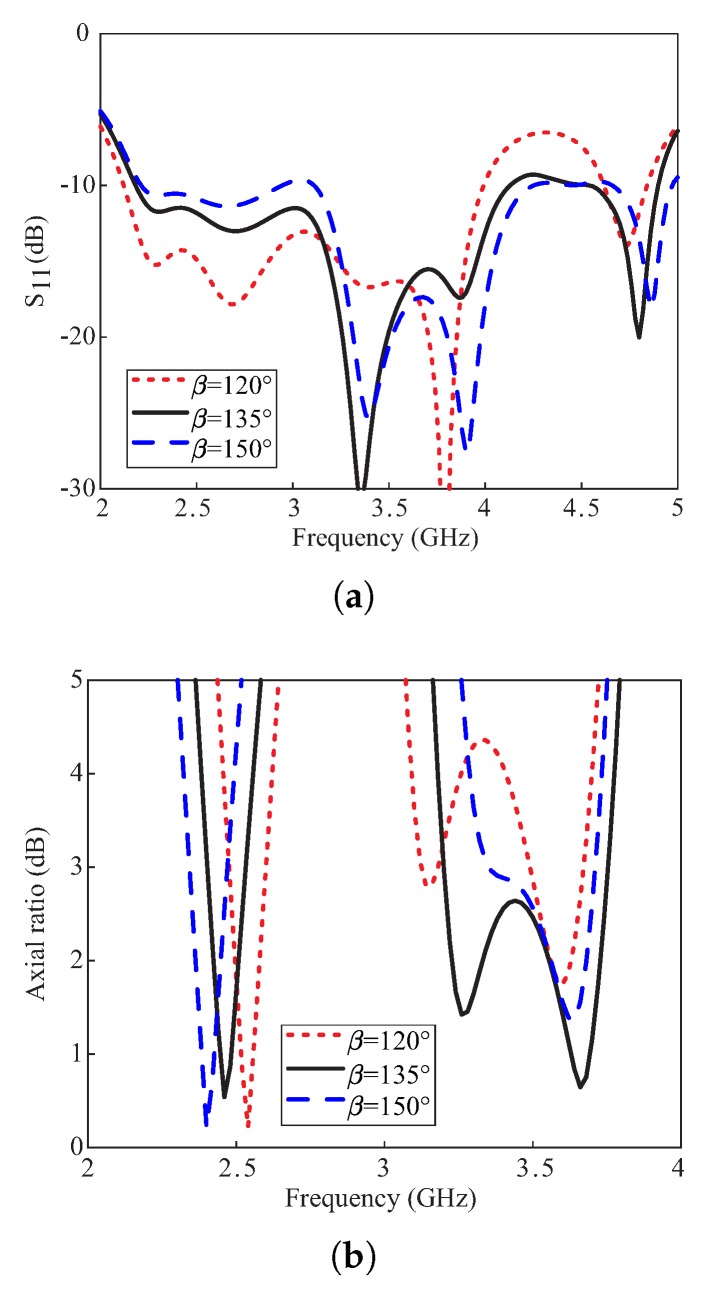
Simulated effects of varying the rotation angle β of the long arm of the DR: (**a**) Reflection coefficients; (**b**) Axial ratios.

Figure 7 depicts the simulated effects of variations in rotation angle β of the long arm of the Y-shaped DR on the reflection coefficients and ARs. It is observed from Figure 7a that the reflection coefficients around 3 GHz alter with this variation and wide −10 dB IBWs are obtained for β≥ 135°. From Figure 7b, the ARs at the lower CP band are not much affected by this variation. However, a wide 3 dB ARBW at the upper band is only obtained with the medium and long arms of the DR are orthogonal to each other (β−α = 90°). Therefore, the value of β = 135° is selected.

## 4. Measurement Results and Discussion

**Figure 8:**
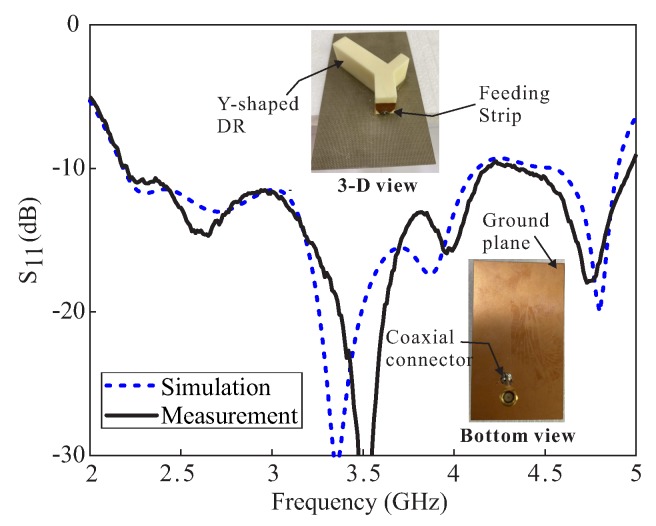
Comparative simulated and measured reflection coefficients of the proposed DRA.

**Figure 9:**
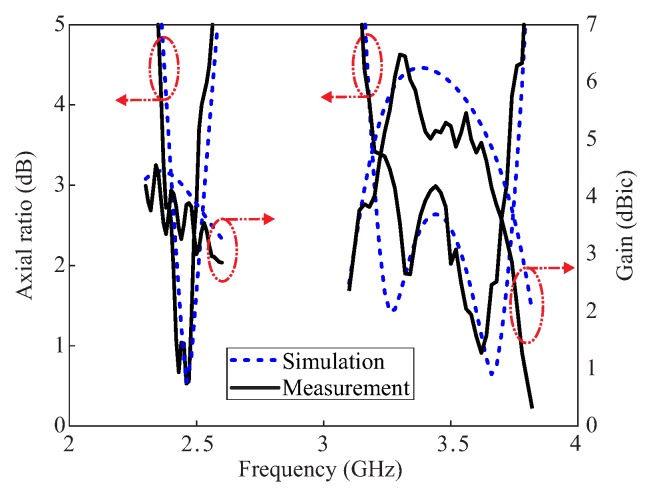
Comparative simulated and measured ARs and Gains of the proposed DRA.

**Figure 10:**
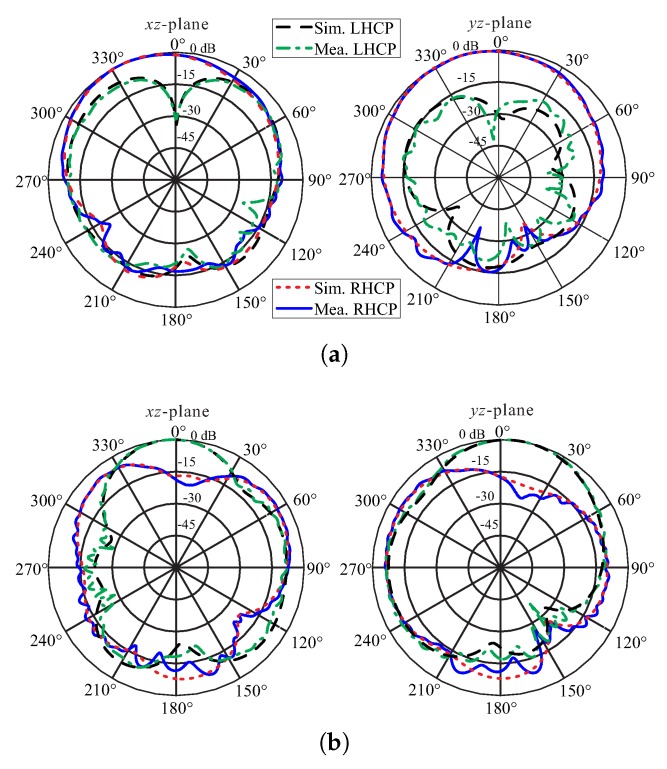
Normalized simulated and measured radiation patterns of the proposed DRA: (**a**) 2.46 GHz; (**b**) 3.5 GHz.

**Figure 11:**
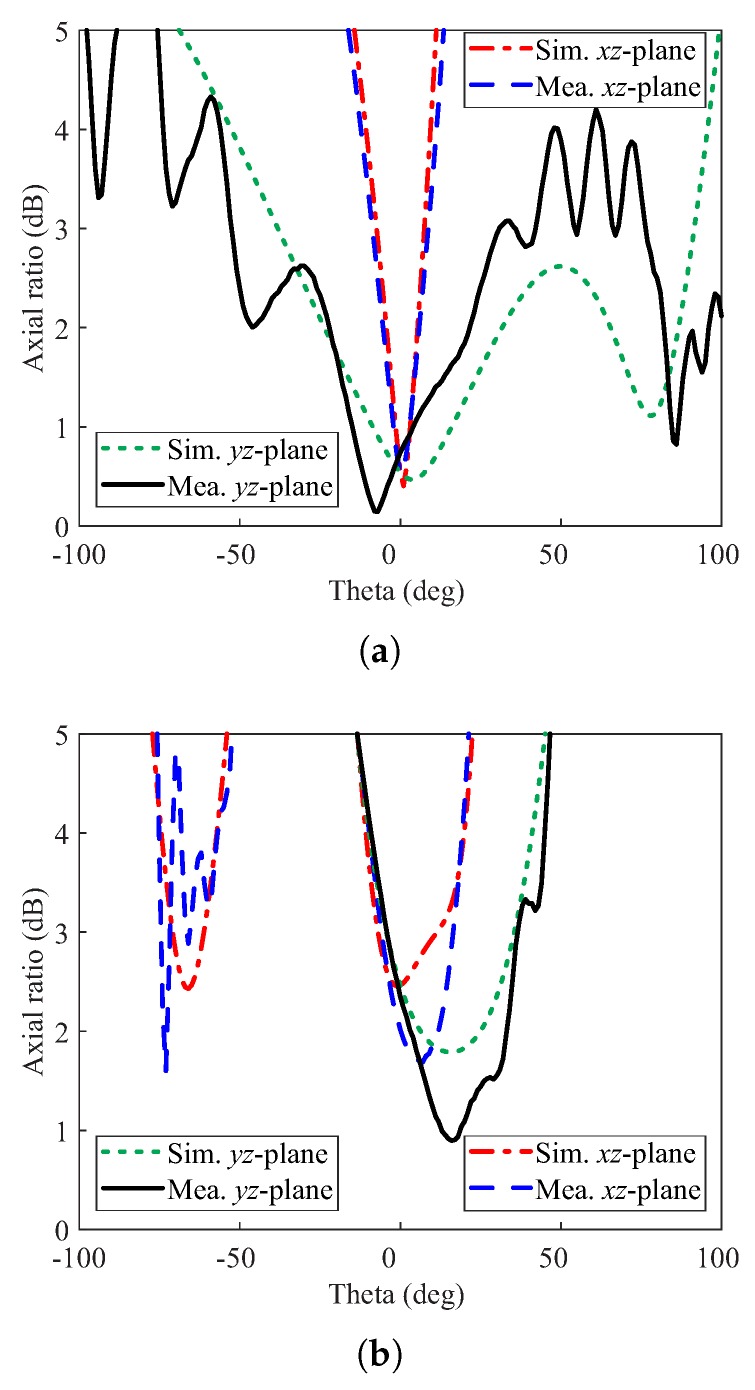
Simulated and measured axial ratios versus theta of the proposed DRA: (**a**) 2.46 GHz; (**b**) 3.5 GHz.

Figure 8 contains a photograph of the fabricated prototype along with a comparison of the simulated and measured reflections coefficients. The measurement is carried out by using the Agilent E5071B network analyzer. The measured result shows −10 dB IBW of 62.07% (2.2–4.18 GHz) as compared to the simulated value of 61.80% (2.18–4.13 GHz). The far-field measurements of the fabricated version for the +*z*-direction were performed in an anechoic chamber. The comparison of the simulated and measured ARs and gains are depicted in Figure 9. The simulated/measured AR values are noted as 5.27 % (2.4–2.53 GHz)/ 4.92% (2.38–2.5 GHz) and 15.83% (3.2–3.75 GHz)/12.64% (3.26–3.70 GHz) for the lower and upper bands, respectively. The peak gains at the lower and upper bands are found to be 4.11 dBic and 6.48 dBic, respectively. From the figure, there is a reasonable similarity between the simulated and measured AR results. The deviation of the measured results from the simulation is related to a slight misalignment between the Tx antenna and antenna under test (AUT), and fabrication tolerances. Nonetheless, the measured AR values cover all 12 WLAN channels (2.401–2.495 GHz) at the lower CP band and WiMAX frequency range (3.4–3.69 GHz) at the upper CP band. The comparison of the normalized radiation patterns for two planes (xz- and yz-planes) at the lower and upper CP bands are plotted in Figure 10. For broadside direction, the measured co-polarized gain is noted as 3.86 dBic and 5.23 dBic at 2.46 GHz and 3.5 GHz, respectively (see Figure 9). Figure 11 depicts the AR versus theta graphs on xz- and yz-planes at 2.46 GHz and 3.5 GHz. From Figure 11a, the measured theta span for AR ≤ 3 dB is found to be ≈17.5° (−9–8.5°) and ≈83.5° (−52.5–31°) as compared to the simulated values of ≈15° (−7.5–7.5°) and ≈130.5° (−39–91.5°) for the xz- and yz-planes, respectively. At 3.5 GHz, the 3 dB CP beamwidths from the measured results are ≈22.5° (−6–16.5°) and ≈41° (−4–37°) as compared to the simulated values of ≈19° (−7–12°) and ≈40° (−4–36°) for the xz- and yz-planes, respectively.

Table 2 tabulates a performance comparison of the proposed work to some of the earlier dual-band CP DRAs [1,2,3,4,5,6,7]. Considering −10 dB IBW, only the proposed work has a wide-band response that makes it favorable for UWB applications, while a dual-band response is noted for all of the referenced works. Compared with [1], the proposed work has a wider AR bandwidth at the upper band. The proposed work has wider ARBWs at both bands and higher gain at the upper band as compared with [3,5]. In comparison with [2,7], the proposed work has higher gain at both bands and wider ARBW only at the upper band. The proposed work performs better concerning the 3 dB ARBW and gain at the upper CP band as compared to [4,6].

## 5. Conclusions

In this paper, a novel dual-band CP DRA is proposed for WLAN and WiMAX applications. The circular polarization at the bands of interest is obtained due to the excitation of a fundamental (TE${}_{111}$) and second-order (TE${}_{211}$) modes. To verify the simulation, a prototype is experimentally verified in terms of −10 dB IBW and far-field performance. The measured data shows a wide −10 dB IBW of 62.07%(2.2–4.18) and the existence of two CP bands with 3 dB ARBWs of 4.92% (2.38–2.50 GHz) and 12.64% (3.26–3.7 GHz). The measured normalized radiation patterns show that the LHCP level is more than 27 dB higher than RHCP at the lower band, while it exceeds the RHCP by more than 17 dB in the upper band at the broadside direction. The CP bands of the proposed antenna entirely cover WLAN and WiMAX frequency ranges and can be adopted in the transceiver design. 

## Figures and Tables

**Table 1:** Optimized geometric parameters of the proposed antenna.

Parameters	Values	Parameters	Values
h	20.5 mm	$l_{3}$	17.96 mm
$f_{w}$	5 mm	$l_{4}$	5.27 mm
$f_{1}$	7 mm	$w_{a}$	4.75 mm
$f_{2}$	7 mm	$w_{b}$	4.5 mm
$g_{l}$	120 mm	$w_{c}$	3.5 mm
$g_{w}$	70 mm	$w_{1}$	11.5 mm
$g_{x}$	20.5 mm	$w_{2}$	7 mm
$g_{y}$	32 mm	$w_{3}$	12 mm
$l_{1}$	40.13 mm	α	45°
$l_{2}$	13.25 mm	β	135°

**Table 2:** Performance comparison of the proposed DRA with earlier dual-band CP DRAs. LB: lower band; UB: upper band.

Ref. No.	($|S_{11}|<-$10 dB) LB/UB (GHz) [%]	(AR < 3 dB) LB/UB (GHz) [%]	Gain (dBic) LB/UB
[1]	(1.45–1.87) [25.3]/(2.1–3) [35.3]	(1.53–1.63) [6.3]/(2.40–2.49) [3.68]	6.09/8.49
[2] (Results for *p* = 3)	(3.29–3.92) [17.47]/(4.52–6.05) [28.94]	(3.29–3.76) [13.33]/(4.55–4.92) [7.81]	3/< 0
[3]	(3.4–3.58) [5.16]/(5.1–5.9) [14.55]	(3.46–3.54) [2.29]/(5.18–5.34) [3.04]	≈5.1/≈5.1
[4]	(5.51–7.46) [30.07]/(11.42–12.37) [7.98]	(6.08–7.43) [19.98]/(11.84–12.21) [3.07]	4.85/6.38
[5]	(1.22–1.36) [11.4]/(1.50–1.64) [8.4]	(1.26–1.28) [2.1]/(1.54–1.57) [2.2]	5.5/4.5
[6]	(4.57–5.79) [23.55]/(8.05–9.2) [13.33]	(4.75–5.5) [14.84]/(8.55–9.18) [7.11]	4.3/5.8
[7]	(2.77–3.66) [27.7/(3.93–4.28) [8.5]	(3.08–3.6) [15.7]/(4.05–4.3) [6]	2.3/4.7
Proposed work	2.2–4.18 [62.07]	(2.38–2.5) [4.92]/(3.26–3.7) [12.64]	4.11/6.48
